# Identification of Tumor Mutation Burden, Microsatellite Instability, and Somatic Copy Number Alteration Derived Nine Gene Signatures to Predict Clinical Outcomes in STAD

**DOI:** 10.3389/fmolb.2022.793403

**Published:** 2022-04-11

**Authors:** Chuanzhi Chen, Yi Chen, Xin Jin, Yongfeng Ding, Junjie Jiang, Haohao Wang, Yan Yang, Wu Lin, Xiangliu Chen, Yingying Huang, Lisong Teng

**Affiliations:** ^1^ Department of Surgical Oncology, The First Affiliated Hospital, School of Medicine, Zhejiang University, Hangzhou, China; ^2^ Department of Oncology-Pathology, Karolinska Institute, Solna, Sweden; ^3^ Department of Breast Surgery, Zhuji Affiliated Hospital of Shaoxing University, Zhuji, China

**Keywords:** gastric cancer, immunotherapy response, predictive model, copy number alteration, tumor mutation burden, microsatellite instability

## Abstract

Genomic features, including tumor mutation burden (TMB), microsatellite instability (MSI), and somatic copy number alteration (SCNA), had been demonstrated to be involved with the tumor microenvironment (TME) and outcome of gastric cancer (GC). We obtained profiles of TMB, MSI, and SCNA by processing 405 GC data from The Cancer Genome Atlas (TCGA) and then conducted a comprehensive analysis though “iClusterPlus.” A total of two subgroups were generated, with distinguished prognosis, somatic mutation burden, copy number changes, and immune landscape. We revealed that Cluster1 was marked by a better prognosis, accompanied by higher TMB, MSIsensor score, TMEscore, and lower SCNA burden. Based on these clusters, we screened 196 differentially expressed genes (DEGs), which were subsequently projected into univariate Cox survival analysis. We constructed a 9-gene immune risk score (IRS) model using LASSO-penalized logistic regression. Moreover, the prognostic prediction of IRS was verified by receiver operating characteristic (ROC) curve analysis and nomogram plot. Another independent Gene Expression Omnibus (GEO) contained specimens from 109 GC patients was designed as an external validation. Our works suggested that the 9‐gene‐signature prediction model, which was derived from TMB, MSI, and SCNA, was a promising predictive tool for clinical outcomes in GC patients. This novel methodology may help clinicians uncover the underlying mechanisms and guide future treatment strategies.

## Introduction

Immune checkpoint blockade (ICB) therapy targeting programmed cell death protein 1 (PD-1) and cytotoxic T lymphocyte antigen 4 was tolerated with manageable toxicities and promising antitumor effect in patients with GC (Aggelis et al., 2018). However, for low response rates to single-agent anti-PD-1 therapy or anti-CTL4 treatment in unselected patients, single-agent immunotherapy would not be an appropriate treatment of patients with operable GC (Xie et al., 2020). Moreover, previous ICB studies have shown that response rates to immunotherapy vary widely in GC, ranging from 10 to 26% (Zeng et al., 2021). Thus, finding optimal biomarkers to identify potential responders to immunotherapy remains an urgent priority.

Cancer genomic characteristics had a high profile due to their key role in ICB resistance and their potential in biomarker prediction. Currently, several biomarkers had been used to predict ICB responses despite some limitations. For example, tumor mutation burden (TMB) high was well documented to contribute to therapeutic response to ICB, especially in patients with melanoma and non-small cell lung cancer (Rizvi et al., 2015; Van Allen et al., 2015). TMB high patients had a higher chance of mobilizing the immune system to augment responding to ICB. Similarly, it was reported that microsatellite instability (MSI) high GC led to somatic mutation accumulation as well as therapy-induced immunosurveillance (van Velzen et al., 2020). In addition, previous studies suggested that the copy number instability score and the genome instability number, calculated based on somatic copy number alteration (SCNA), can serve as an early indicator of immune checkpoint inhibitor response versus progression (Weiss et al., 2017; Jensen et al., 2019). However, due to limitations such as measuring barriers and the absence of tumor markers, these biomarkers were rarely detected in patients with GC in practice. Early assessment of response to immunotherapy remained a current unmet clinical and scientific need to discern therapy response and tumor progression. Therefore, an integrated approach incorporating various molecular features would be warranted to understand the unifying perspectives of the mechanisms underlying ICB resistance and identify subgroups of GC patients with immune microenvironments.

Recently, the integration of multiple omics profiles with high-throughput molecular analysis had been a major focus for the discovery of multiple cancer subgroups. Deep learning approaches allowed for a systematic understanding of genomic, proteomic, biochemical, metabolic, and epigenetic processes. The most commonly used integration tools include “mixOmics,” “tRanslatome,” “R.JIVE,” and “iClusterPlus”. First, “mixOmics” was a powerful framework with four kinds of datasets (metabolomics, phenomics, cell wall proteomics, and transcriptomics) (Durufle et al., 2021). Then, a deep neural network named “tRanslatome” was proposed which can predict the protein structure from input amino acid sequences but not for disordered proteins (Du et al., 2021). Later on, “R.JIVE” was proposed by O’Connell, an algorithm for exploratory dimension reduction, which could decompose the transcriptomic and proteomic data (O'Connell and Lock, 2016). Finally, “iClusterPlus” shows high compatibility and accuracy in subgroup identification, containing discrete and continuous parameters that are derived from genomic, transcriptomic, and epigenomic features (Mo et al., 2013).

In the present study, integrative clustering of three genomic datasets including TMB, SCNA, and MSI were used to investigate subgroups of GC through “iClusterPlus” software. We further estimated the TME infiltration patterns of stomach adenocarcinoma (STAD) from TCGA and GEO data and systematically analyzed the clusters’ relationship with genomic characteristics and clinical features in GC. We incorporated the TME infiltration evaluation into an immune risk score (IRS) to predict ICB therapeutic efficacy and survival outcomes from tumor genomic data. Depicting the immune landscape features of GC, more importantly, contributes to interpret the immunotherapy response of GC and provide new strategies for cancer treatment.

## Materials and Methods

### Data Source

TCGA-STAD gene expression data (n = 440), mutation annotation format (MAF) (n = 440), somatic copy number data (n = 405), and clinical data of the corresponding patient (n = 444) were obtained from cBioPortal (http://cbioportal.org/) and UCSC Xena (http://xena.ucsc.edu/) websites. Finally, 405 patients with complete data were screened for subsequent analysis. The clinical characteristics of involved patients are displayed in Table 1. Furthermore, we conducted the transcriptome sequencing data in both raw read counts and fragments per kilobase per million mapped reads (FPKM) values, and counts data were applied to DEG analyses, whereas FPKM data were calculated for microsatellite instability (MSI) evaluation. The GSE26901 data from the National Center for Biotechnology Information (NCBI) was derived as an independent validation cohort (n = 109). All genomic coordinates for TCGA data and GEO data in analyses of our study were based on the GRCh37 genome reference sequence (Jensen et al., 2017).

**TABLE 1:** Clinicopathological information of the TCGA-STAD dataset.

Clinicopathological traits	Type	Patient number
Gender	Male	261
	Female	144
Age	≤60	130
	>60	275
Grade	G1	12
	G2	143
	G3	241
	Gx	9
StageT	T1	21
	T2	86
	T3	179
	T4	111
	Tx	8
StageN	N1	121
	N2	108
	N3	77
	N4	81
	Nx	18
StageM	M0	366
	M1	23
	Mx	16
Stage	I	58
	II	128
	III	174
	IV	39
	X	6

### SCNA Data Acquisition and Processing

The peak regions of recurrent DNA copy number alteration including amplification and deletion were delineated by GISTIC2 algorithm (Mermel et al., 2011). Subsequently, we converted copy number alteration into binary form and defined them as SCNA genomic features. We categorized SCNA events, as reported, according to each patient’s aberration status of GISTIC results: −2, homozygous loss; −1, hemizygous deletion; 0, diploid; 1, low-level gain; and 2, high-level amplification. High-level amplifications and homozygous loss in the peak region were defined as copy number change, with at least 50% of genes displaying an amplification or deletion (Xie et al., 2020). To obtain the binary description matrix about the SCNA feature, we assigned feature changes as 1 and no feature changes as 0.

SCNA scores were calculated using masked copy number segment profiles from the UCSC Xena platform, which is defined as the ratio of copy number alteration (tumor/normal) and normalized by fragment length after log2 transformation.

### Modified TMB (mTMB) Data Acquisition and Processing

We defined mTMB as the total number of unique genes with mutations in each patient. Only seven types of mutations in this gene were considered as mTMB event: Frame_Shift_Del, Translation_Start_Site, Frame_Shift_Ins, Splice_Site, Non-stop_Mutation, Non-sense_Mutation, and Missense_Mutation. After removing no functional relevance mutations, we merged the MAF data of TCGA-STAD. Then, the low-frequency mutated genes were filtered through a cutoff value (a certain gene mutation occurred in 1% of the total number of samples). As a result, we extracted 1932 high-frequency mutated genes in 405 patients, and the binary description matrix of mTMB feature was used with subsequent calls.

TMB burden was computed by the total number of somatic mutations per Mb in each sample. Since 38 Mb is usually taken in terms of the length of human exons, the TMB burden was equal to the total mutation frequency/38 (Schumacher et al., 2015).

### MSI Data Acquisition and Processing

MSIsensor-pro algorithm of the Linux operating system was used to investigate the MSI traits of TCGA-STAD data at the microsatellite transcription level (Jia et al., 2020). We selected and calculated the most frequently altered microsatellite sites to construct the binary MSI feature based on the somatic mutation status of each sample. Then, we computed the MSIsensor score under the default parameters by a sample matrix. We further distinguished MSI high (MSIsensor score≥10) samples from MSI low or microsatellite stability (MSS) (MSIsensor score <10) samples, according to the previous research (Niu et al., 2014; Abida et al., 2019). Finally, 1 represents MSI and 0 represents MSI low/MSS to obtain a binary matrix of the MSI event.

### Genomics Variation Data Integration

We constructed a comprehensive data of 2,024 genome variant characteristics, including 54 copy number gains, 37 copy number losses, 1 MSI, and 1,932 genes. Subsequently, we characterized the SCNA, mTMB, and MSI traits in a binary form to delineate whether corresponding genomic alteration occurred in each patient. In detail, 1 indicated the presence of genomic changes while 0 indicated the absence of genomic changes in this data, which formed the sample matrix with three binary signatures.

### Clustering and Survival Analysis

In our genomic variation profiles description matrix, the columns represent various samples, while rows represent the corresponding genomic signatures. In total, 405 valid samples were classified by “iClusterPlus,” a comprehensive clustering method in the R package (Mo et al., 2018a). With default parameters, different numbers of categories are cycled (k = 1–5). Finally, the optimal classification result was calculated with the highest percent of explained variation and best Bayesian information criteria, that is, k = 1 and Cluster = 2 (Supplementary Figure. S1A). We selected the top quartile features based on LASSO coefficient estimates (prob = 0.75). Thus, only values greater than the upper quartile were considered to contribute significantly to the classification.

### Mutation and Copy-Number Aberration Analysis

Mutation analysis was performed both in Cluster1 and Cluster2 based on the “maftools” package in R. The default arguments were set to analyze the MAF of the TCGA-STAD dataset in each cluster. The mutation results were directly visualized by “oncoplot” function of the “maftools” R package. The analysis of CNAs was performed with GISTIC2 on the Linux system. The specific “-conf” parameter was set to 0.95, and top 10 significant copy number alteration areas were displayed in both clusters with “gisticOncoPlot” function. The G-score across all chromosomes was visualized based on the frequency of the CNAs and the average amplitude in the log ratio.

### Immune Cell Infiltration Analysis

The cancer immune infiltration profiles in the two clusters were compared based on the gene expression data of TCGA-STAD. The proportion of various immune cells and proportion of each sample were computed through “CIBERSORT” software under the default parameters. Differences of the immune cell landscape between Cluster1 and Cluster2 were investigated by “boxplot”. Additionally, the well-established “TMEscore” was used to analyze the difference in immune efficacy between the two clusters (Zeng et al., 2019).

### Differentially Expressed Gene Analysis

Based on the TCGA-STAD gene expression data (counts), we used the “DESeq2” package in R to screen out the DEGs between the two clusters. The significance threshold for DEGs was set to abs (log2FC) > 1 and *p* < 0.05.

### Immune Risk Score Model Construction

The normalized expression data of the DEGs were subsequently converted into binary fashion by comparing the median value of each gene in all samples. After combining with clinical data, DEGs were further selected in univariate cox analysis using “coxph” function of the “survival” package in R. With default parameters and significance (*p* < 0.05), we carried out the hub genes as independent prognostic factors. The “glmnet” package was used to perform LASSO-penalized regression on the samples and corresponding DEGs. The arguments used in LASSO-penalized regression are alpha = 1, nlambda = 100, and *p* < 0.05 was considered as the significant threshold. By “coef” function, we got the *Y*-intercept and hazard rate (HR) score of each gene. Then, HR score, *Y*-intercept, and corresponding hub gene expression profiles were used to measure the IRS value of all samples.

### Statistical Analysis

The unpaired Student’s t-test was developed to estimate the comparison between two normally distributed variables. In contrast, non-normally distributed variables were measured by the Wilcoxon rank-sum test. In order to compare more than two groups, Kruskal–Wallis tests and ANOVA were used as the non-parametric method and the parametric method, respectively. Two-sided Fisher’s exact tests were used to analyze contingency data. The Kaplan–Meier method was used to offer a visual representation of predicted survival curves for each cluster data with “ggplot2” and “survminer” packages. Area under the curve (AUC), sensitivity, and specificity were depicted by the “pROC” package. All statistical analyses in this study were performed on R version 4.0.4 (https://www.r-project.org/), and *p*-value < 0.01 indicated a statistically significant threshold.

## Results

### Comprehensive Genomic Variation Traits to Identify Two GC Classifications

**FIGURE 1:**
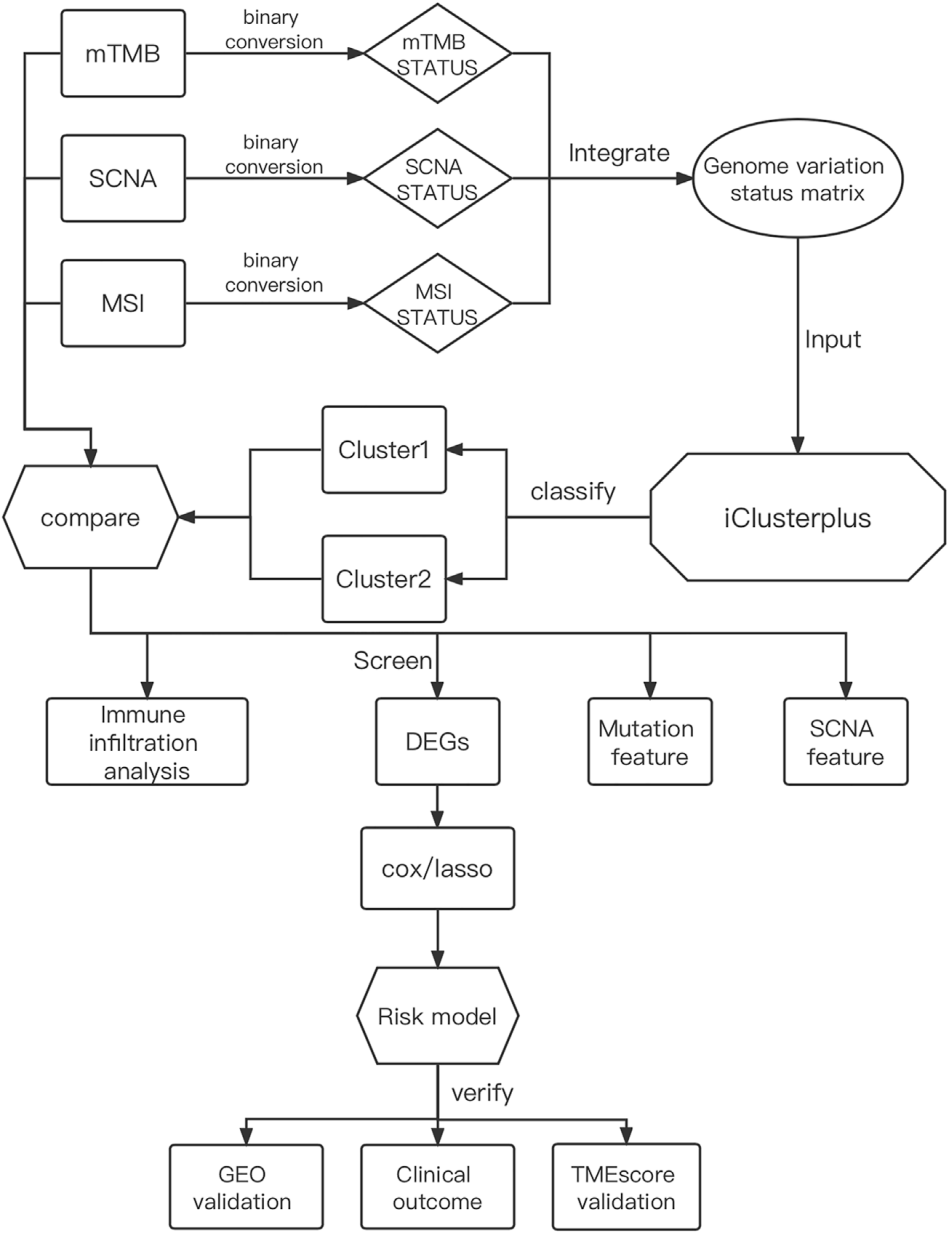
Comprehensive workflow of our study for constructing the risk model in GC, including downloading and processing and analysis.

**FIGURE 2:**
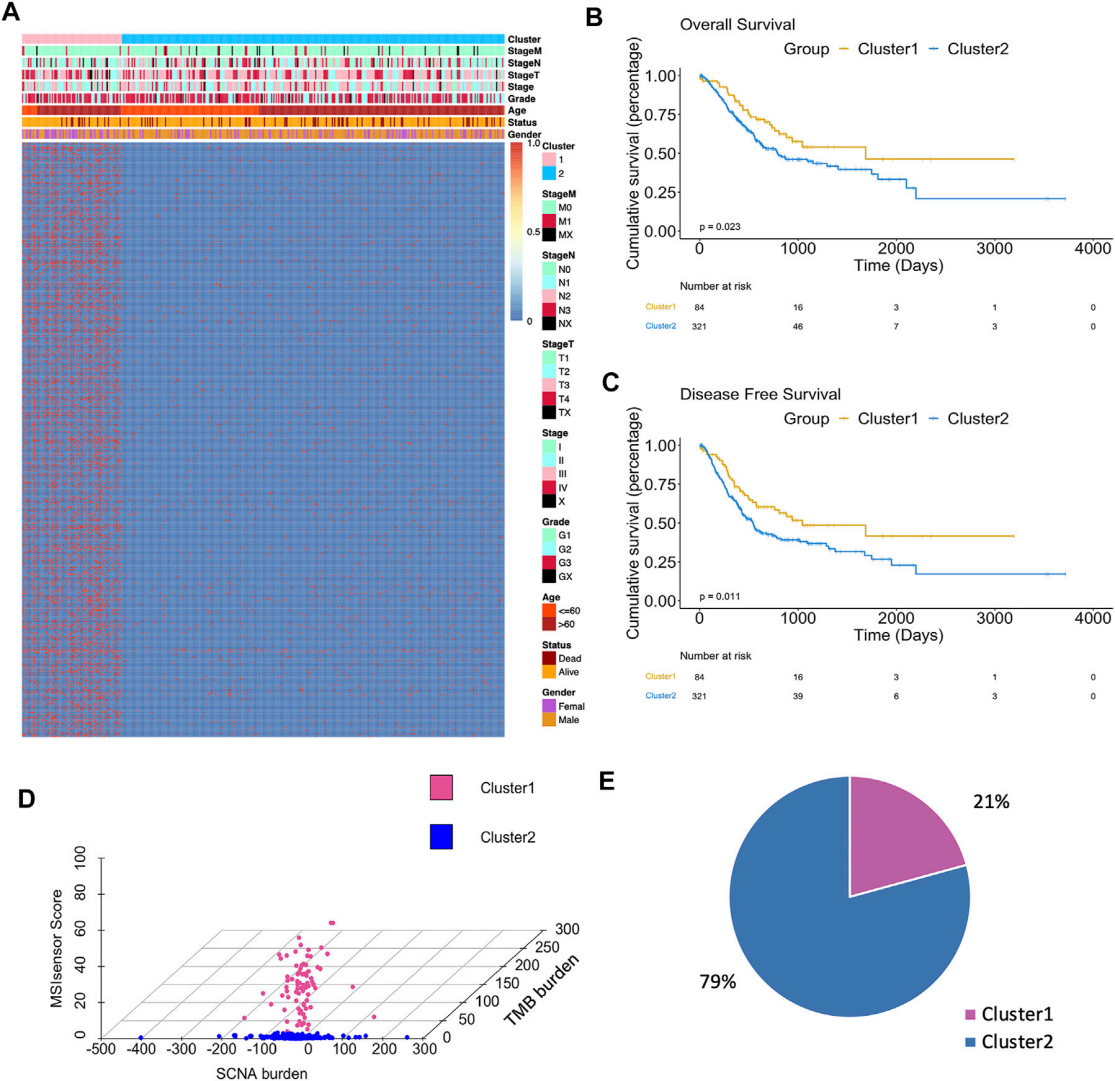
Differences in clinicopathological traits and genomic features between two clusters. **(A)** Heatmap showed clusters of 500 genomic mutation profiles. Sample annotations show the clinicopathological characteristics. **(B)** Kaplan–Meier plots showed the OS in patients in Cluster1 and Cluster2 (*p* < 0.05). **(C)** Kaplan–Meier plots showed the DFS in patients in Cluster1 and Cluster2 (*p* < 0.05). **(D)** 3D plot showed the difference in samples between two clusters. **(E)** Sample percentage of the two clusters.

The integrated design workflow in our study is shown in Figure 1. According to SCNA patterns, MAF data, and MSI signature of the TCGA-STAD, we meticulously characterized the three genomic statuses. After integrating the three genomic statuses (a total of 2,024), all the 405 STAD samples were divided into Cluster1 and Cluster2 based on “iClusterPlus” (Figure 2A and Supplementary Figure S1A). It was worth noting that Cluster1 had a higher proportion of older patients (>60 years) than Cluster2. In contrast, Cluster2 was the main cluster of deaths cases and more likely to gather higher level stageM, stage, and grade on STAD, indicating that the traits of genomic change were distinctly associated with the cancer malignancy. In terms of gene mutations, the samples with more gene mutations were obviously clustered in Cluster1. Additionally, the heatmap of MSI (top panel), SCNA (middle panel), and mutation (bottom panel) for the three-cluster solution is shown in Supplementary Figure S1B. To further compare the clinical value of our clusters, we performed Kaplan–Meier (KM) plots to explore the outcomes of the two subgroups. Interestingly and noteworthy, we found that patients in Cluster1 showed significantly longer overall survival (OS) and disease-free survival (DFS) than Cluster2 (Figures 2B,C). Subsequently, we used the “scatterplot3d” package to visualize the MSIsensor score, SCNA burden, and TMB burden across the samples, and the results demonstrated that Cluster1 and Cluster2 can be well distinguished (Figure 2D). The take-home message of the number of samples is that the percentages of Cluster1 and Cluster2 cases are 21 and 79%, respectively (Figure 2E).

### Different Signatures of MSI, SCNA, and mTMB in Two Clusters

**FIGURE 3:**
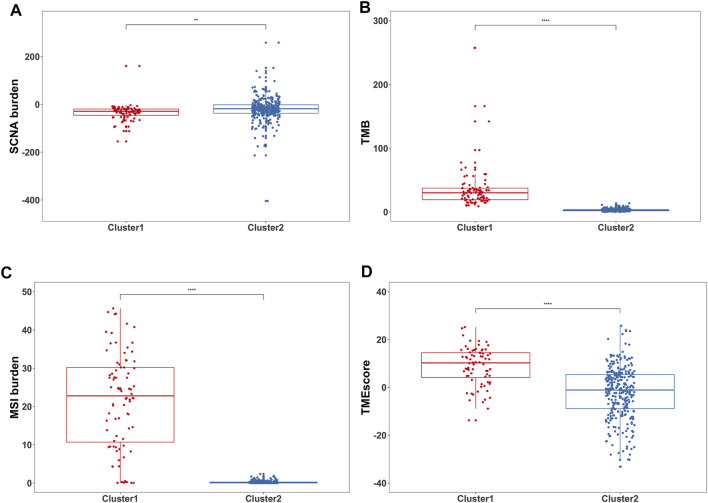
Differences in genomic variations and immune measurement between the two subgroups. **(A)** Box plots showed the SCNA burden between two clusters **p < 0.01. **(B)** Box plots showed the TMB between two clusters ****p < 0.0001. **(C)** Box plots showed the MSI burden between two clusters ****p < 0.0001. **(D)** Box plots showed the TMEscore between two clusters ****p < 0.0001.

Next, the relevant quantitative indicators of three genomic characteristics including MSI, SCNA, and TMB burden were analyzed between the two clusters. In terms of the SCNA burden, Cluster1 was relatively lower than Cluster2 (Figure 3A). The significant differences in TMB burden and MSI burden were also shown between Cluster1 and Cluster2. Specifically, Cluster1 harbored markedly higher TMB burden and MSI burden than Cluster2 in TCGA-STAD (Figures 3B,C). Our results suggested that Cluster1 tends to be associated with better prognosis in GC patients, with the underlying assumption that SCNA low, TMB high, and MSI were more likely to benefit from ICB, which was consistent with previous reports (Liu et al., 2019; Lu et al., 2020). To assess the relative level of immune infiltration in the subgroups, as a commonly accepted quantifiable indicator (Zeng et al., 2019; Huang et al., 2020; Zhang et al., 2021a; Jiang et al., 2021), TMEscore was conducted to compare the difference between the two groups. Intriguingly, Cluster1 was observed with significantly higher TMEscore than Cluster2, which represented more abundant cancer-infiltrating immune cells (Figure 3D).

### Differences in Somatic Mutations and CNAs Between Two Clusters

**FIGURE 4:**
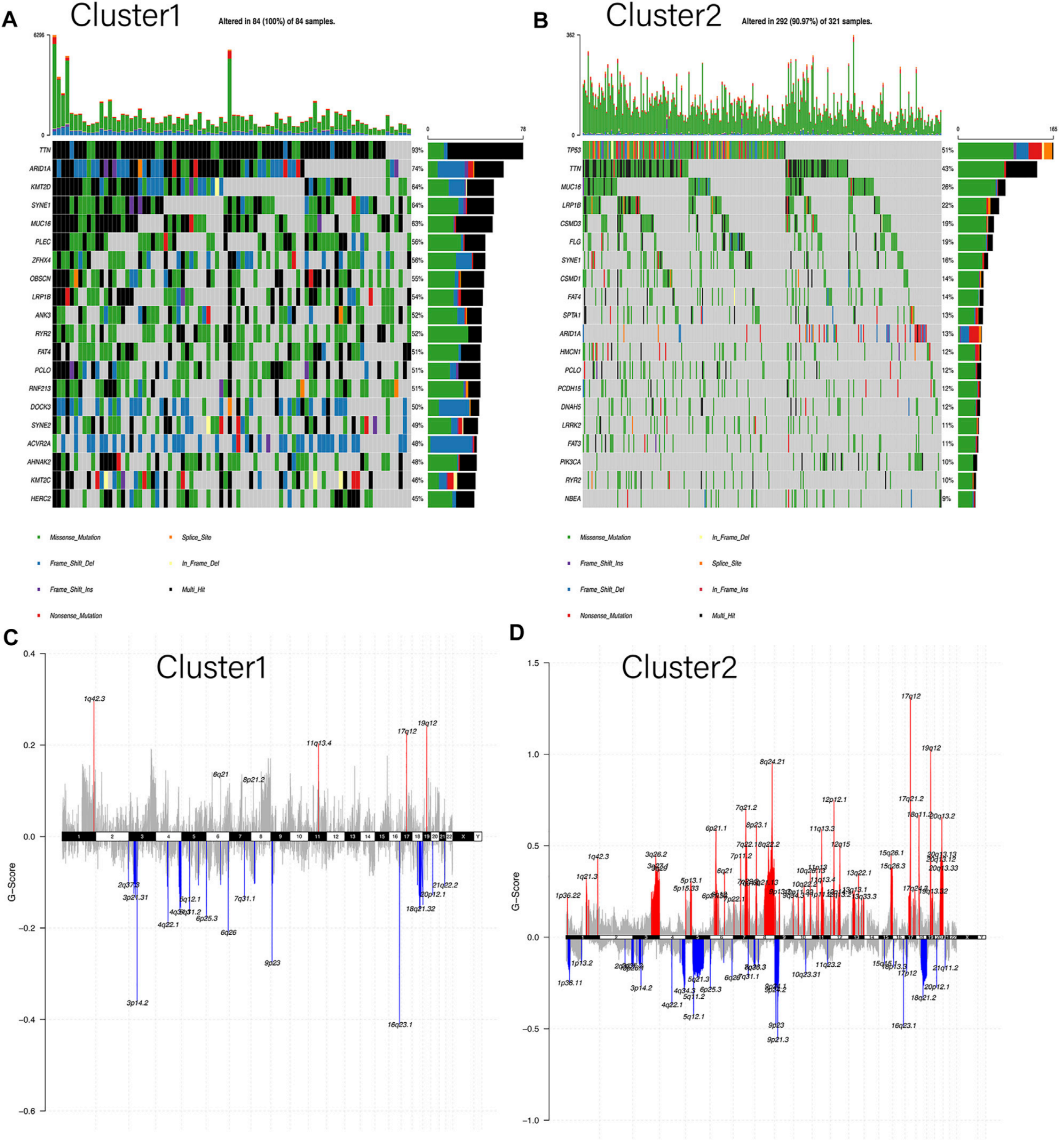
Differences in somatic mutation and copy number alteration between the two subgroups. **(A,B)** Oncoplot indicated the top 20 mutated genes in both Cluster1 and Cluster2, respectively. Different colors represent different mutation types. **(C,D)** Cumulative CNA regions for Cluster1 and Cluster2, respectively. Amplification was represented in red color, and deletion was represented in blue color.

To elucidate the differences between two subgroups, the somatic mutation landscapes in a waterfall plot were displayed (Figures 4A,B). Our work clarified the total mutation load in each sample and sorted the top 20 genes by mutation frequency in each subgroup. Notably, as the *Y*-axis represented, the mutation load of Cluster1 was distinctly higher than that of Cluster2, in accordance with the results in Figure 3B. Moreover, only seven genes in the top 20 mutant genes were shared between the two subgroups: *TTN*, *MUC16*, *LRP1B*, *SYNE1*, *FAT4*, *PCLO*, and *ARID1A*. However, these shared genes in Cluster1 were more likely to have “multi-hits mutation” instead of “missen mutation” compared to Cluster2. Meanwhile, the CNA landscapes of the two clusters were shown in chromosomal alterations via the G-score (Figures 4C,D). Interestingly, Cluster2 had a higher copy number variation frequency on chromosomes 6, 7, and 8 than Cluster1. On the whole, the copy number region between the two clusters demonstrated that Cluster2 had a higher genome-wide amplification and deletion than Cluster1, consistent with the results in Figure 3A. In addition, heat maps were used to identify the top 10 CNAs in each cluster, in which the main changes of Cluster1 were deletion (green) while in Cluster2 were amplification (red) (Supplementary Figure S2A). Also, the proportion of CNAs detected in Cluster1 (23–42%) is significantly lower than that in Cluster2 (57–77%). Overall, these results indicated that mutations and CNAs on both clusters were significantly different.

### TME Landscape of Two GC Subgroups

**FIGURE 5:**
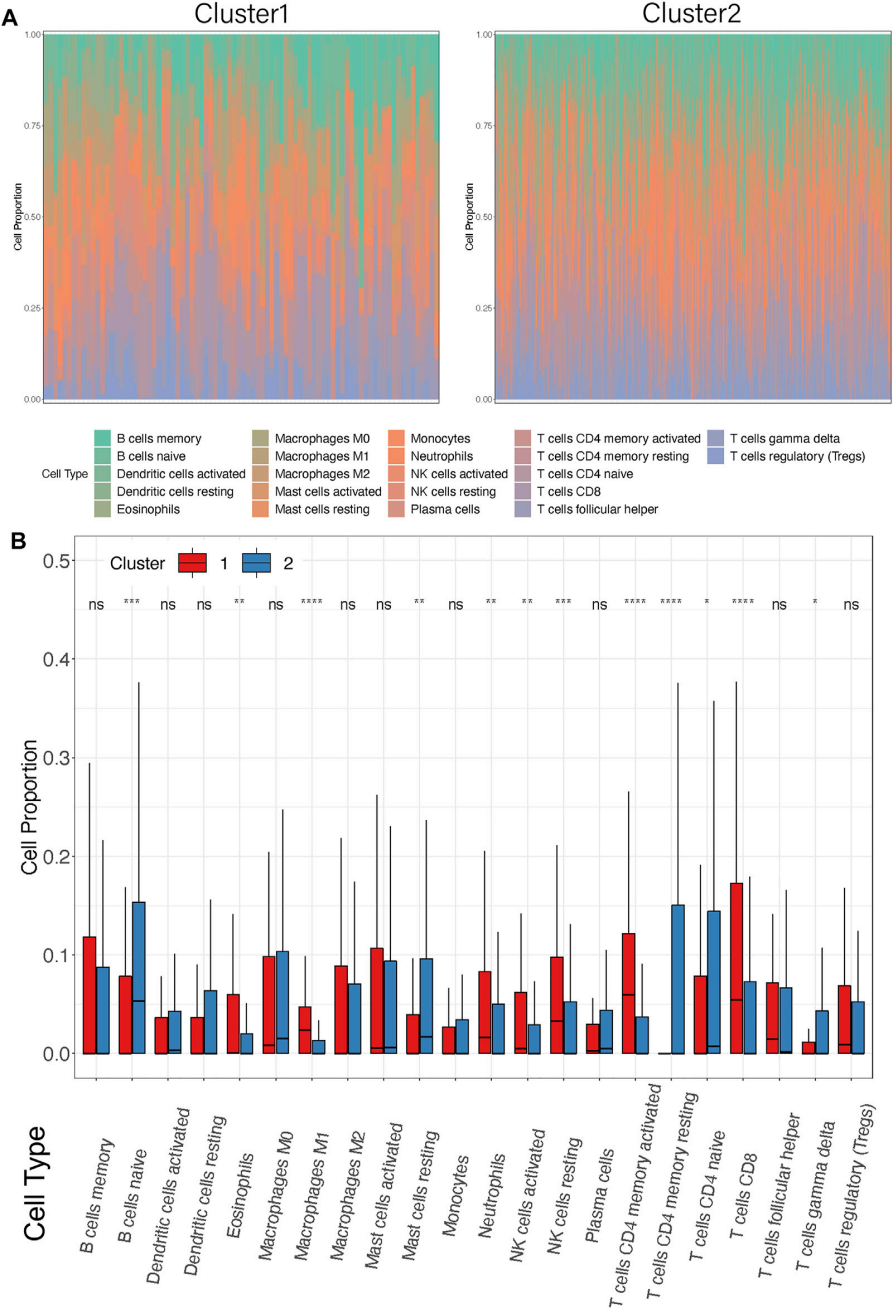
Differences in immune cell infiltrations between the two clusters. **(A)** Distribution of 22 infiltrating immune cell subtypes between the two clusters in each patient. Different colors represent different immune cell types. Each bar on the *X*-axis represents a STAD sample. *Y*-axis represents the percentage of different cell types in each sample. **(B)** Box plots showed the infiltration of immune cell proportion between Cluster1 and Cluster2. *X*-axis represents the different immune cell subtypes. *Y*-axis represents the cell proportion in each cluster. *p < 0.05, **p < 0.01, ***p < 0.001, ****p < 0.0001.

To investigate the TME between the two subgroups, CIBERSORT analysis was carried out to display the abundance of 22 immune cell types (Figure 5A). In addition, activation status and enumeration of the 12 immune cells were significantly discrepant, as shown in Figure 5B. The ratio of eosinophils (*p* = 0.0024), macrophages M1 (*p* < 0.0001), neutrophils (*p* = 0.0002), NK cells active (*p* = 0.0078), NK cells resting (*p* = 0.0008), t cells CD4 memory active (*p* < 0.0001), and T cells CD8 (*p* < 0.0001) was markedly higher in Cluster1 than that in Cluster2. On the other side, compared with Cluster1, the infiltration of B cells naïve (*p* = 0.0002), mast cells resting (*p* = 0.0084), t cells CD4 memory resting (*p* < 0.0001), t cells CD4 naïve (*p* = 0.0141), and t cells gamma delta (*p* = 0.0165) were much extensive in Cluster2. Moreover, we selected seven immune cell types which showed high infiltration in more than half of the samples (Supplementary Figure S2B). It is noteworthy that similar results were observed in Cluster2, with increased B cells naive and mast cells resting. The rejection and immune dysfunction levels were further validated by TMEscore (Figure 3D). The aforementioned results suggested that Cluster1 was more likely to trigger antitumor immunity rather than Cluster2.

### Construction of the IRS Model

**FIGURE 6:**
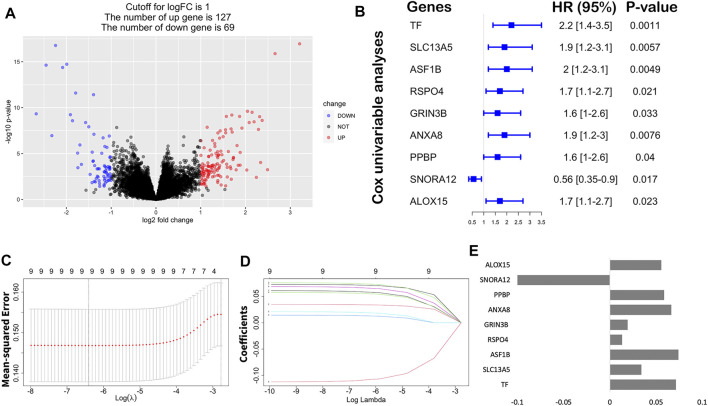
Construction of the immune risk score model. **(A)** Volcano plot indicated the association between log2 fold change and *p*-value in DEGs, abs (log2FC) > 1 and *p* < 0.05. **(B)** Univariate analysis of nine significant genes with OS (*p* < 0.05). **(C)** Correlation between log(λ) and the mean-squared error in the LASSO Cox regression model. **(D)** Correlation between log(λ) and the coefficients of relevant in the LASSO Cox regression model. **(E)** Coefficients of each independent variable in the LASSO Cox regression model.

To further identify the differences in the transcription level between the two clusters of STAD, a total of 196 DEGs were detected by the “DESeq2” package (Table 2; Figure 6A). Under the cutoff of *p* < 0.05 and |log2FC|>1, we screened 127 upregulated genes and 69 downregulated genes in Cluster2. Then, univariate cox analysis was conducted to investigate the 196 DEGs.

**TABLE 2:** DEGs between two genomic variation clusters (top20, Cluster1 vs. Cluster2).

Gene	baseMean	log2FoldChange	lfcSE	Stat	Pvalue	Padj	Change
*CALB1*	55.7570737	3.20704641	0.3745911	8.56145924	1.11E-17	1.41E-13	Up
*FABP4*	112.55467	−2.2424426	0.26338141	−8.5140503	1.68E-17	1.41E-13	Down
*SLC6A10P*	163.583951	2.65889326	0.32140159	8.27280672	1.31E-16	7.30E-13	Up
*LEP*	7.57683434	−1.9914675	0.25052301	−7.9492398	1.88E-15	7.86E-12	Down
*CD300LG*	5.88931295	−2.4530037	0.30967674	−7.9211752	2.35E-15	7.88E-12	Down
*PLIN1*	31.0236722	−2.0838749	0.26551582	−7.8484018	4.21E-15	1.18E-11	Down
*WISP2*	30.2571827	−1.7930524	0.25611821	−7.0008783	2.54E-12	6.09E-09	Down
*AVPR2*	12.0243545	−1.3938919	0.20080671	−6.9414605	3.88E-12	8.12E-09	Down
*ANXA8*	92.398049	2.04381023	0.32305158	6.32657553	2.51E-10	4.66E-07	Up
*CHGB*	154.368293	2.14618156	0.3413288	6.28772477	3.22E-10	5.40E-07	Up
*TUSC5*	11.0175881	−2.6739849	0.42933709	−6.2281713	4.72E-10	7.19E-07	Down
*CAPN14*	12.2466455	−1.9103772	0.30843019	−6.1938723	5.87E-10	8.19E-07	Down
*COL11A2*	66.8601521	1.69388149	0.27408034	6.18023708	6.40E-10	8.25E-07	Up
*TGM1*	44.3421124	1.57217566	0.25667972	6.1250483	9.07E-10	1.02E-06	Up
*PPP2R2C*	132.681633	1.86737944	0.30492448	6.12407195	9.12E-10	1.02E-06	Up
*RHOXF2B*	6.86820936	2.31485118	0.3786162	6.11397818	9.72E-10	1.02E-06	Up
*KIF1A*	104.350953	2.3710194	0.39752231	5.96449389	2.45E-09	2.42E-06	Up
*THRSP*	4.23481843	−1.8631954	0.31305487	−5.9516577	2.65E-09	2.47E-06	Down
*NOTUM*	427.531768	2.19037069	0.37084952	5.90635974	3.50E-09	3.08E-06	Up
*GTSF1*	42.543178	−2.08174551	0.3542695	−5.876164	4.20E-09	3.36E-06	Up

**FIGURE 7:**
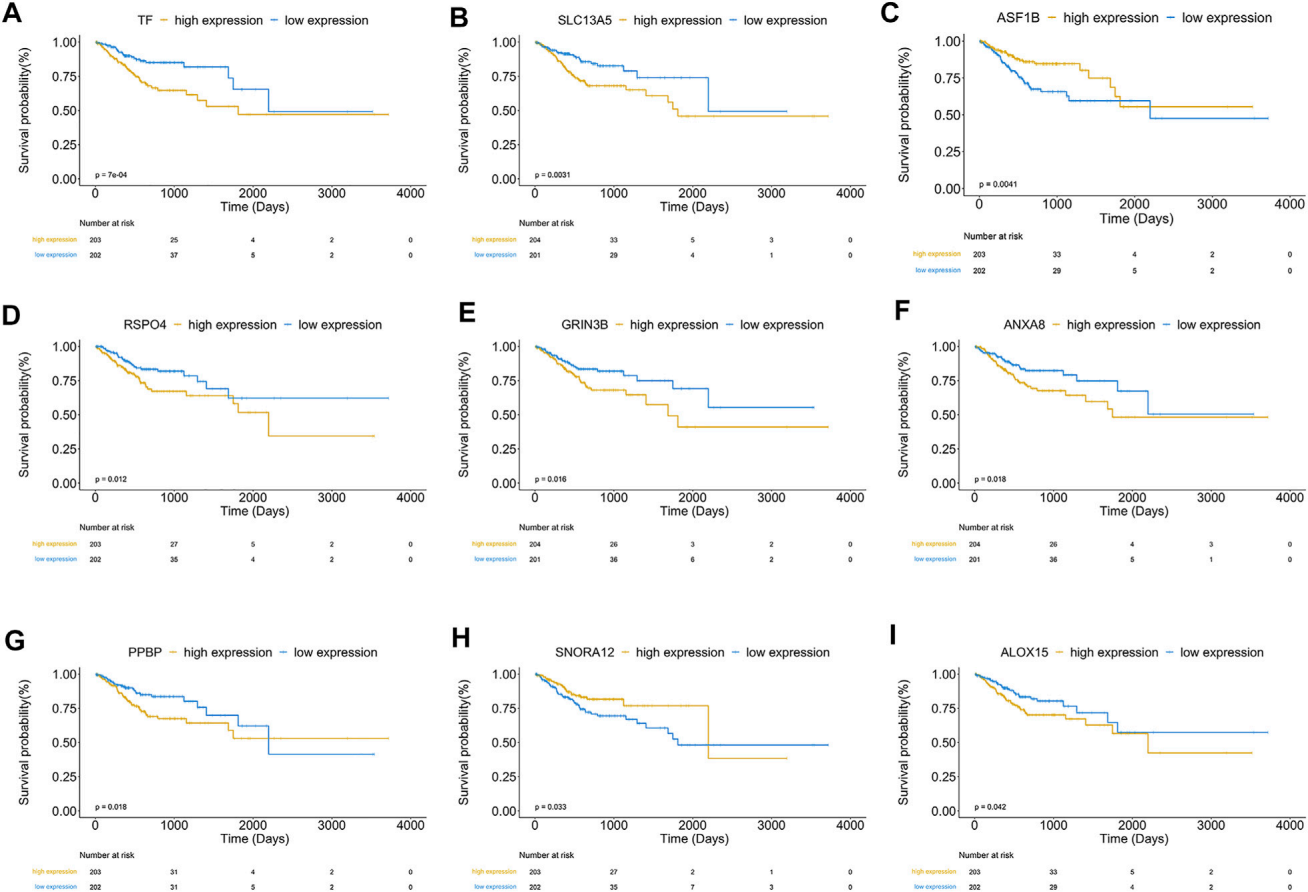
**(A–I)** showed KM plots in patients with high and low expression of nine significant genes, respectively, based on the median value. *p* < 0.05.

Finally, nine hub genes were found to be closely correlated with OS (Table 3; *p* < 0.05), in which *SNORA12* were revealed to be the protective factors, while *TF, SLC13A5, ASF1B, RSPO4, GRIN3B, ANXA8, PPBP,* and *ALOX15* were the risk factors (Figure 6B). Subsequently, we performed LASSO-penalized multivariate cox modeling across 100 simulation replications and constructed an optimal model with nine coefficients, i.e., IRS model (Figures 6B,C,E). The IRS formula used for each sample is shown in Table 4. The predicted value of the model was compared with the actual event in a boxplot (Supplementary Figure S3). Then, the gene expression for the nine genes across all the samples was divided into high expression and low expression by the median value, and corresponding survival differences were shown on KM plots (Figure 7). These nine genes were significantly correlated with OS in STAD, indicating the reliability of our IRS model.

**TABLE 3:** Univariate Cox results of significant DEGs.

Characteristic	HR	Lower	Upper	Combine	p.value
*TF*	2.2	1.4	3.5	2.2 [1.4–3.5]	0.0011
*SLC13A5*	1.9	1.2	3.1	1.9 [1.2–3.1]	0.0057
*ASF1B*	2	1.2	3.1	2 [1.2–3.1]	0.0049
*RSPO4*	1.7	1.1	2.7	1.7 [1.1–2.7]	0.021
*GRIN3B*	1.6	1	2.6	1.6 [1–2.6]	0.033
*ANXA8*	1.9	1.2	3	1.9 [1.2–3]	0.0076
*PPBP*	1.6	1	2.6	1.6 [1–2.6]	0.04
*SNORA12*	0.56	0.35	0.9	0.56 [0.35–0.9]	0.017
*ALOX15*	1.7	1.1	2.7	1.7 [1.1–2.7]	0.023

**TABLE 4:** IRS formula for each sample is calculated as follows:

Immune Risk Score = 0.049 + 0.071*TF (Exp)+ 0.034* SLC13A5 (Exp) + 0.074*ASF1B(Exp) +0.013*RSPO4 (Exp) +0.019* GRIN3B (Exp) +0.066*ANXA8 (Exp) +0.059*PPBP(Exp) +0.0288*NPY(Exp) −0.109*SNORA12(Exp) +0.056*ALOX15 (Exp).

### Evaluating the Discriminatory Power of the IRS Model and External Validation

**FIGURE 8:**
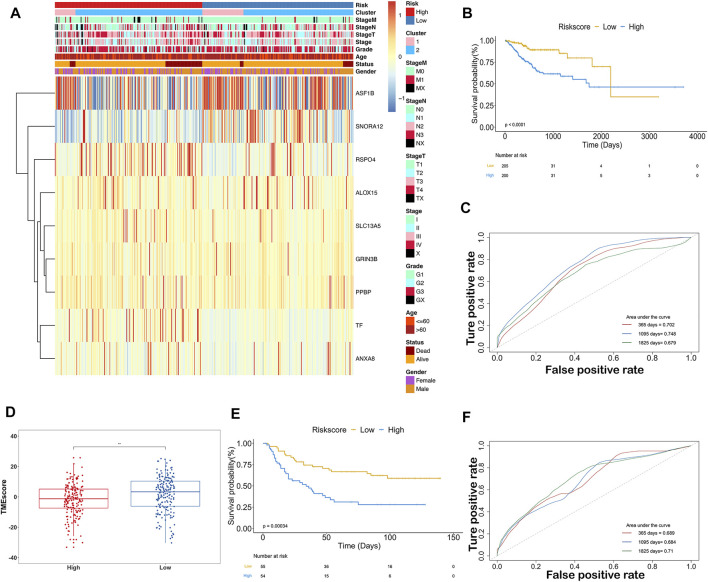
Predictive ability of the ISR model in TCGA and GEO databases. **(A)** Expression of 9-gene and clinicopathological features in each patient (TCGA-STAD). Heatmap showed 9-gene expression profiles in high-IRS and low-IRS groups, based on the median value. Sample annotations show the clinicopathological characteristics and clusters. **(B)** KM plots showed the OS in the training group with high IRS and low IRS (*p* < 0.0001). **(C)** Prognostic values of IRS in the training group in 1-, 3-, and 5-year OS with AUC = 0.702, 0.748, and 0.679, respectively. **(D)** Box plots showed the difference of TMEscore between high IRS and low IRS. **(E)** KM plots showed the OS in the validation group with high IRS and low IRS (*p* < 0.001). **(F)** Prognostic values of IRS in the validation group in 1-, 3-, and 5-year OS with AUC = 0.689, 0.684, and 0.71, respectively.

Samples were classified into high-IRS and low-IRS groups based on the median value of IRS. A comprehensive heatmap was developed to display the distribution of clinical characteristics in TCGA-STAD data (Figure 8A). Interestingly, the occurrence of cancer-related death was comparatively enriched in the high-IRS group. In addition, Cluster2 and advanced stage were gathered in the high-IRS group. Furthermore, the expression levels of *ASF1B, SNORA12, RSPO4*, and *TF* were visibly different between high- and low-IRS groups. To further evaluate the predictive ability of IRS, the significant differences in OS between high- and low-IRS groups are shown in Figure 8B (*p* < 0.0001). AUC was computed to test the discriminatory powers over 1-year, 3-year, and 5-year outcome (Figure 8C), suggesting a promising prognostic predictive value in our training dataset. In order to measure the value of the IRS model in immunotherapy, the IRS grouping result was compared with TMEscore, and the low-IRS group showed a relatively high TMEscore (Figure 8D). Notably, in the validation cohort (GSE26901), our IRS model suggested a distinct difference between the high- and low-IRS groups in clinical outcomes as well (Figure 8E). Also, the AUC values in 1-year, 3-year, and 5-year were close to 70% (Figure 8F).

### Assessing Predictive Values and Stability on the IRS Model

**FIGURE 9:**
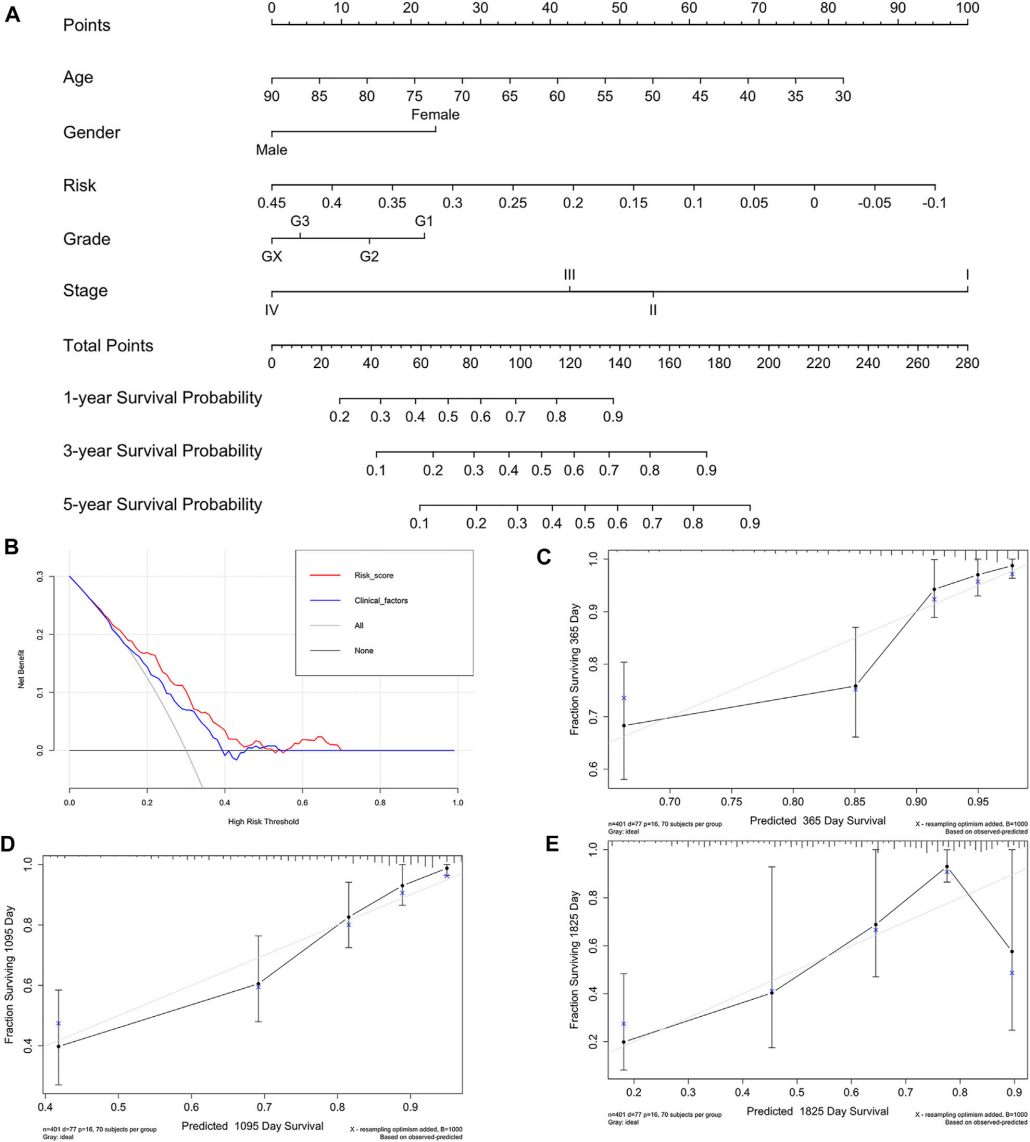
Nomogram of IRS and its stability evaluation. **(A)** Nomogram for predicting survival probability of GC patients in the training group. The total score of clinicopathological features as well as risk score for each sample is located on the “total points” axis, which corresponds to the survival probabilities on the 1-, 3-, and 5-year axes. **(B)** Decision curve for nomogram and clinical factors. **(C–E)** Calibration curves for nomogram in 1-, 3-, and 5-year, respectively.

The indicative clinicopathological features of the samples, including age, gender, grade, stageM, stageN, and stageT, were conducted to test the stability and efficiency of the ISR model. Our results indicated that the ISR showed significant differences between high-IRS and low-IRS samples in all clinical characteristics except in high stageM (Supplementary Figure S4). In addition, we revealed the profile of 22 immune cell infiltrations between high-IRS and low-IRS samples. The proportion of dendritic cells resting and macrophages M1 was significantly higher in low-IRS patients (Supplementary Figure S5), which was consistent with the previous work. We subsequently generated a nomogram calibration plot to combine the clinical factors as well as risk (IRS) to measure the clinical benefits (Figure 9A). Moreover, the decision curve of this prognostic nomogram and the IRS prediction model are displayed in Figure 9B. The nomogram-based 1-year, 3-year, and 5-year OS predictions for GC patients with IRS exhibited superior accuracy (Figures 9C–E).

## Discussion

ICB antibodies had revolutionized the therapeutic landscape in patients with various cancers (Borghaei et al., 2015; Hamanishi et al., 2015; Motzer et al., 2015; Ferris et al., 2016; Rosenberg et al., 2016; Nishino et al., 2017), including advanced GC (Kang et al., 2017). Notably, the PD-L1-combined positive score was widely approved as a predictive biomarker which indicated efficacy of ICB in GC (Kim et al., 2018; Mariathasan et al., 2018). However, these therapeutic responses occurred only in a minority of GC patients, while most GC patients were primarily resistant to ICB (Roh et al., 2017; Fuchs et al., 2018). Previous studies supported the idea that the clinical benefit with ICB in GC was independent of PD-L1-combined positive score positivity (Kang et al., 2017). Thus, the combination of immunotherapy with chemotherapy and angiogenesis inhibitor had been encountering the dilemmas of lacking precise biomarkers. Extensive research studies had proved the predictive ability of SCNA, mTMB, and MSI to therapeutic response or resistance (Rizvi et al., 2015; Van Allen et al., 2015; Le et al., 2017). However, as predictive biomarkers individually, each one of these genomic traits is not stable enough to accurately reflect GC heterogeneity. Here, we comprehensively integrated these ICB-related genomic signatures, i.e., mTMB, MSI, and SCNA to explore pertinent underlying mechanisms.

In our study, with a fully Bayesian latent variable model, we stratified TCGA-STAD into two distinct tumor subtypes according to SCNA, mTMB, and MSI. Intriguingly, each subtype was correlated with a special immune profile highlighting its multidimensional relationship between intrinsic genetic characteristics and TME (Ivey et al., 2016; Krishnan et al., 2018). Currently, TMEscore had been used by many researchers to predict treatment efficacy to ICB as well as to investigate the immune suppressive mechanisms mediated by TME (Qiu et al., 2021; Wei et al., 2020). Based on the 2 GC cohorts, we revealed that our clustering is robust in predicting OS, DFS, and TMEscore (Figure 3). A simple combination of SCNA, mTMB, and MSI or through known benchmarking driver genes was not able to reinforce our understanding of the interplay between the cancer genomic landscape and the host-specific antitumor immune response (Zhao et al., 2020). The advantage of “iClusterPlus” was its sufficient dimension reduction, with unsupervised clustering across all data types, provided the most accurate classification in clinical tumor subtypes, and revealed driver omics features (Abu-Jamous et al., 2017; Mo et al., 2018b). In addition, the distribution of latent variables is more stable, since it was automatically generated by its conditional distribution of visible variables (Menyhárt and Győrffy, 2021). Despite the lack of user-friendliness, this approach greatly met the needs in precision medicine and helped clinicians to diagnose and customize treatments.

To further investigate the differences of the immune microenvironments in the two distinct genomic clusters, CIBERSORT was performed to assess the infiltration of 22 immune cells. It is well established that the polarization of the macrophages to the M1 phenotype could kill the cancer cells and suppress their growth (Menga et al., 2020; Rao et al., 2020). On the other hand, eosinophils had been implicated as antitumor effector cells, whose tumoricidal function was mediated by TNF-α, granzymes, and IL-18 (Reichman et al., 2016; Varricchi et al., 2018). Moreover, neutrophils, NK cells, and T cells had been reported as central communicators in antitumor immunity (Fabian et al., 2020; Chen et al., 2021; Munir et al., 2021). Consistent with our clustering, Cluster1 tended to aggregate these immune cells, activate the immune microenvironment, and had a high potential for response to ICB. In order to explore the gene expression patterns of Cluster1, we screened the DEGs between Cluster1 and Cluster2 and selected the prognostic core markers to construct the prediction model. Inspiringly, our IRS model showed that patients with high IRS had a poorer prognosis and a lower proportion of macrophage M1 infiltration (Supplementary Figures S4 and S5). More importantly, we further used KM plot, AUC, nomogram, and decision curve analysis to validate the predictive value of IRS in calculating the OS probability of GC patients. Merits of our IRS model were primarily attributed to the precise identification of TME activation based on 9 genes, particularly in predominant infiltration of M1 macrophages tumors.

Among these nine key genes, several genes had been reported to be involved in carcinogenesis and tumor progression. For example, *SLC13A5* was a sodium-coupled transporter which was proved to facilitate hepatic energy homeostasis, influence proliferation of hepatocarcinoma, and resist chemotherapeutic agents in hepatocarcinoma cells (Li et al., 2017; Hu et al., 2020). *RSPO4* was a member of the R-spondin family. As WNT signaling activation had been found to overexpress in breast cancer, particularly in triple-negative breast cancer, the role of *RSPO4* involved in GC progression remained unelucidated (Coussy et al., 2017; Park et al., 2018). On a similar note, *ANXA8* had been revealed to be upregulated in various cancers (Gou et al., 2019; Ma et al., 2020; Yuan et al., 2021). The feedback loop between *RA-RARA* and *ANXA8* fostered cancer initiation and progression (Rossetti and Sacchi, 2020). More importantly, the expression levels of *ASF1B* were reported to be associated with TME in STAD (Rossetti and Sacchi, 2020). From a mechanistic point of view, *ASF1B* indirectly regulated *CKS1B* to mediate growth, apoptosis, and cell cycle progression in cancers (Zhang et al., 2021b).

However, due to TME complexity and tumor heterogeneity, not all patients with high IRS would benefit from immunotherapy. This research was limited by the validity of exon-level transcriptomic data from immunotherapy patients. Hence, further work was needed to validate our findings in the prospective cohort of GC patients receiving ICB. In the foreseen future, with the increasing availability of large-scale detection applied to GC patients treated with ICB, a systematic exploration of TME would unveil the mechanisms underlying resistance to immunotherapy.

## Conclusion

In summary, we comprehensively analyzed three genetic features associated with the immune microenvironment and subsequently identified two distinct clusters in GC. We delineated the characteristics of both subgroups from prognosis, mutation burden, copy number changes, and immune profile. Identifying their DEGs followed by screening survival-related genes, nine hub DEGs were finally selected for downstream analysis. We proposed a 9-gene IRS that serves as a biomarker in clinical application, whose predictive value was further validated in an independent GC cohort (GSE26901). Therefore, we developed a nomogram predicting the probability of a patient who will survive GC for 1, 3, and 5 years. Our work provided a new approach to accelerate accurate immunotherapy, which may optimize combination therapy strategies.

## Data Availability

The datasets presented in this study can be found in online repositories. The names of the repository/repositories and accession number(s) can be found in the article/Supplementary Material.

## Supplementary Material

Supplementary Figure S1 Results of iClusterPlus according to three genomic variations. **(A)** Number of clusters vs. percent of explained variation. Cluster selection for sample classification (k = 1-5). **(B)** Heatmap of MSI (top panel), SCNA (middle panel), and mutation (bottom panel) for the three-cluster solution. Rows are genomic features, and columns are samples.

Supplementary Figure S2 Differences in SCNA and main infiltrating immune cell typesbetween two clusters. **(A)** Heatmap plots indicated the top 10 copy number changes in Cluster1 and Cluster2. **(B)** Abundance of seven high infiltrating immune cell types in the two clusters.

Supplementary Figure S3 Box plots showed the predicted value of the IRS model and the actual event.

Supplementary Figure S4 KM plots showed OS in patients with high IRS and low IRS in age, gender, grade, stageM, stageN, and stageT subgroups.

Supplementary Figure S5 Box plots showed differences in the infiltration of 22 immune cells between high-IRS and low-IRS groups.

## Abbreviations

TCGA, The Cancer Genome Atlas; GEO, Gene Expression Omnibus; ICB, immune checkpoint blockade; PD-1, programmed cell death protein 1; GC, gastric cancer; SCNA, somatic copy number alteration; TMB, tumor mutation burden; mTMB, modified TMB; TME, tumor microenvironment; IRS, immune risk score; STAD, stomach adenocarcinoma; MAF, mutation annotation format; DEGs, differentially expressed genes; NCBI, National Center for Biotechnology Information; MSS, microsatellite stability; HR, hazard rate; AUC, area under the curve; KM, Kaplan–Meier; OS, overall survival; DFS, disease-free survival.
