# Supplementary figures and images for: Identification of Tumor Mutation Burden, Microsatellite Instability, and Somatic Copy Number Alteration Derived Nine Gene Signatures to Predict Clinical Outcomes in STAD
Source: Front Mol Biosci. 2022 Apr 11;9:793403. doi: 10.3389/fmolb.2022.793403 (PMC9037630; doi:10.3389/fmolb.2022.793403)

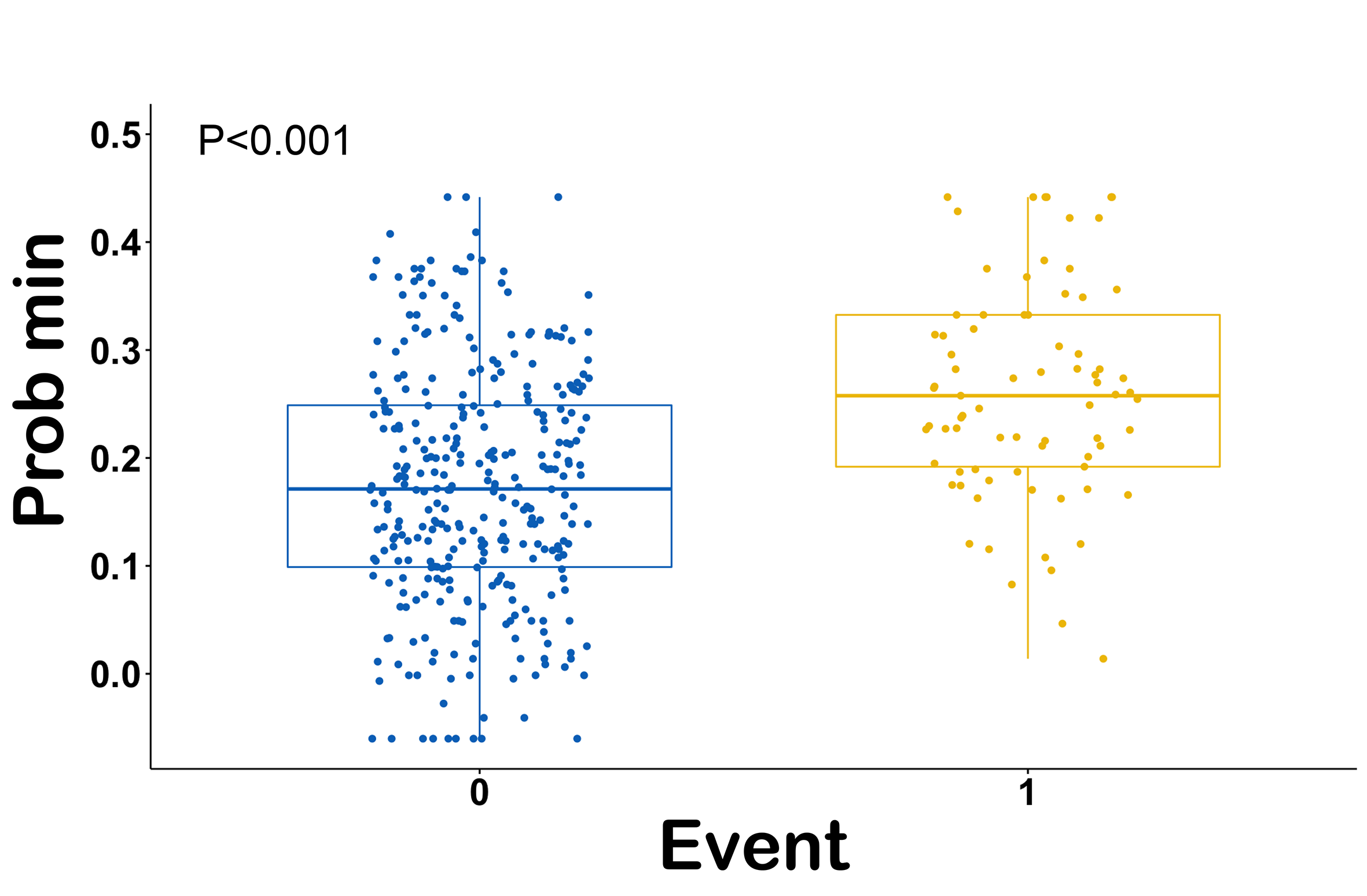

Supplement: Supplementary file 1 [file Image3.tif]

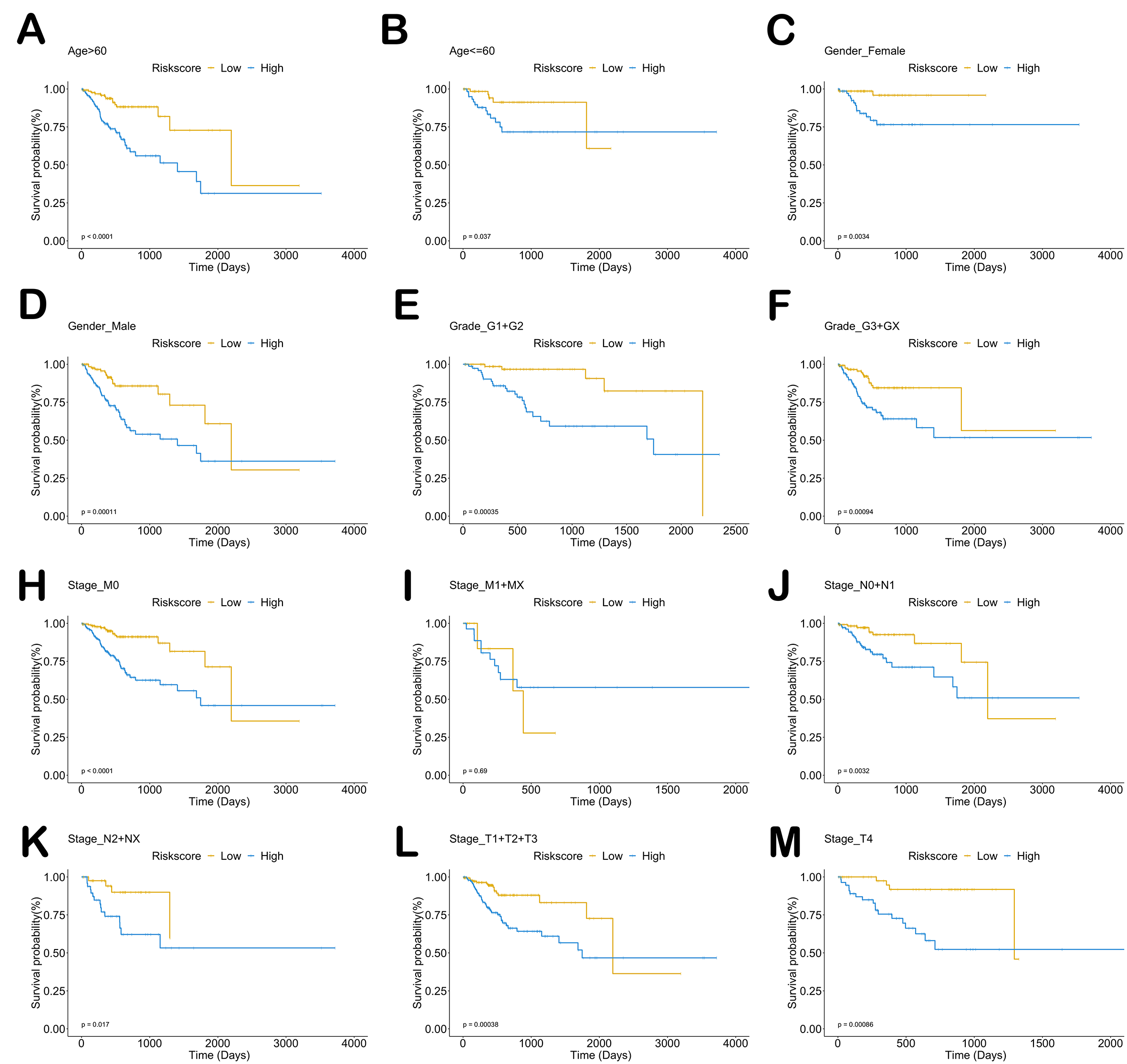

Supplement: Supplementary file 2 [file Image4.tif]

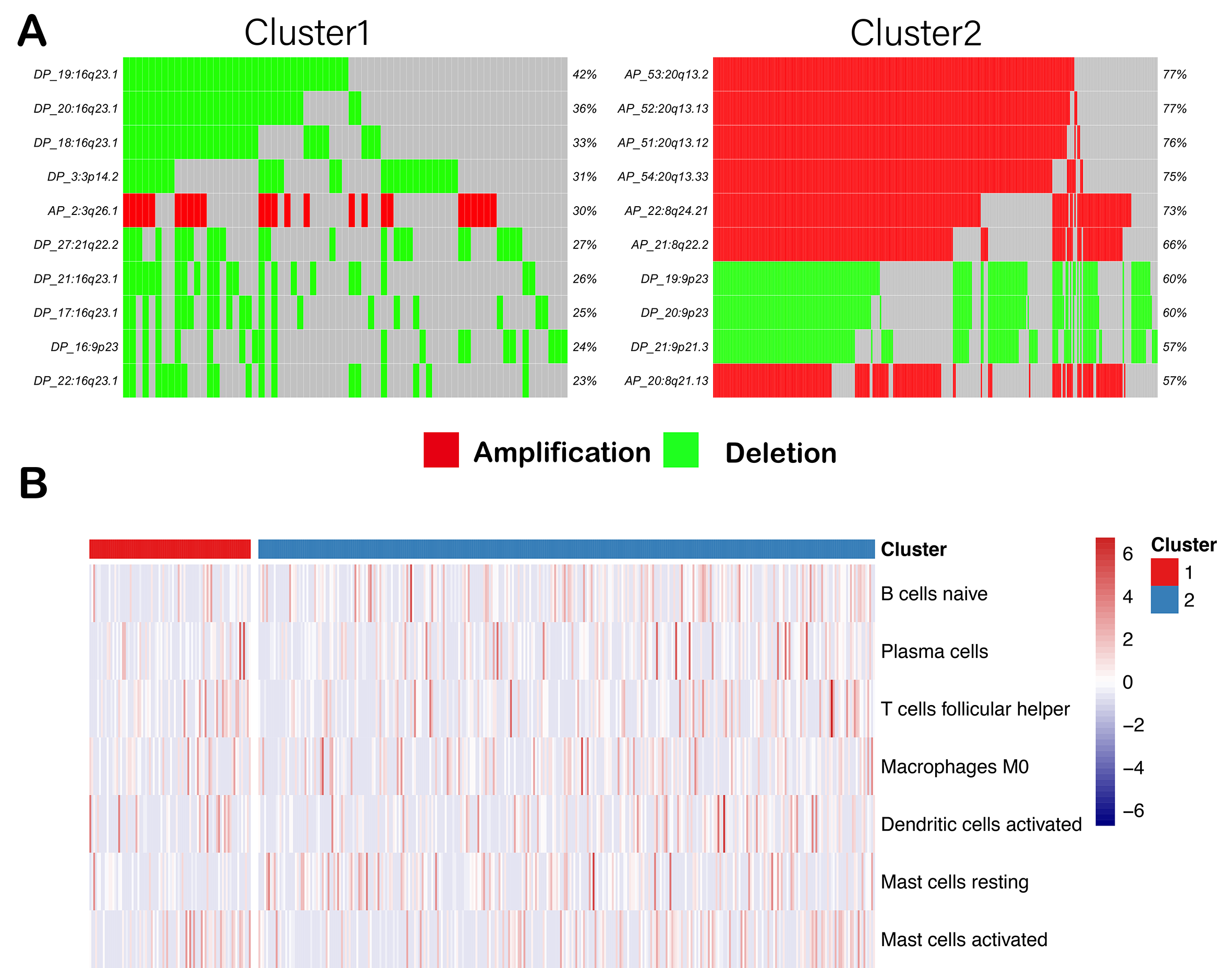

Supplement: Supplementary file 3 [file Image2.tif]

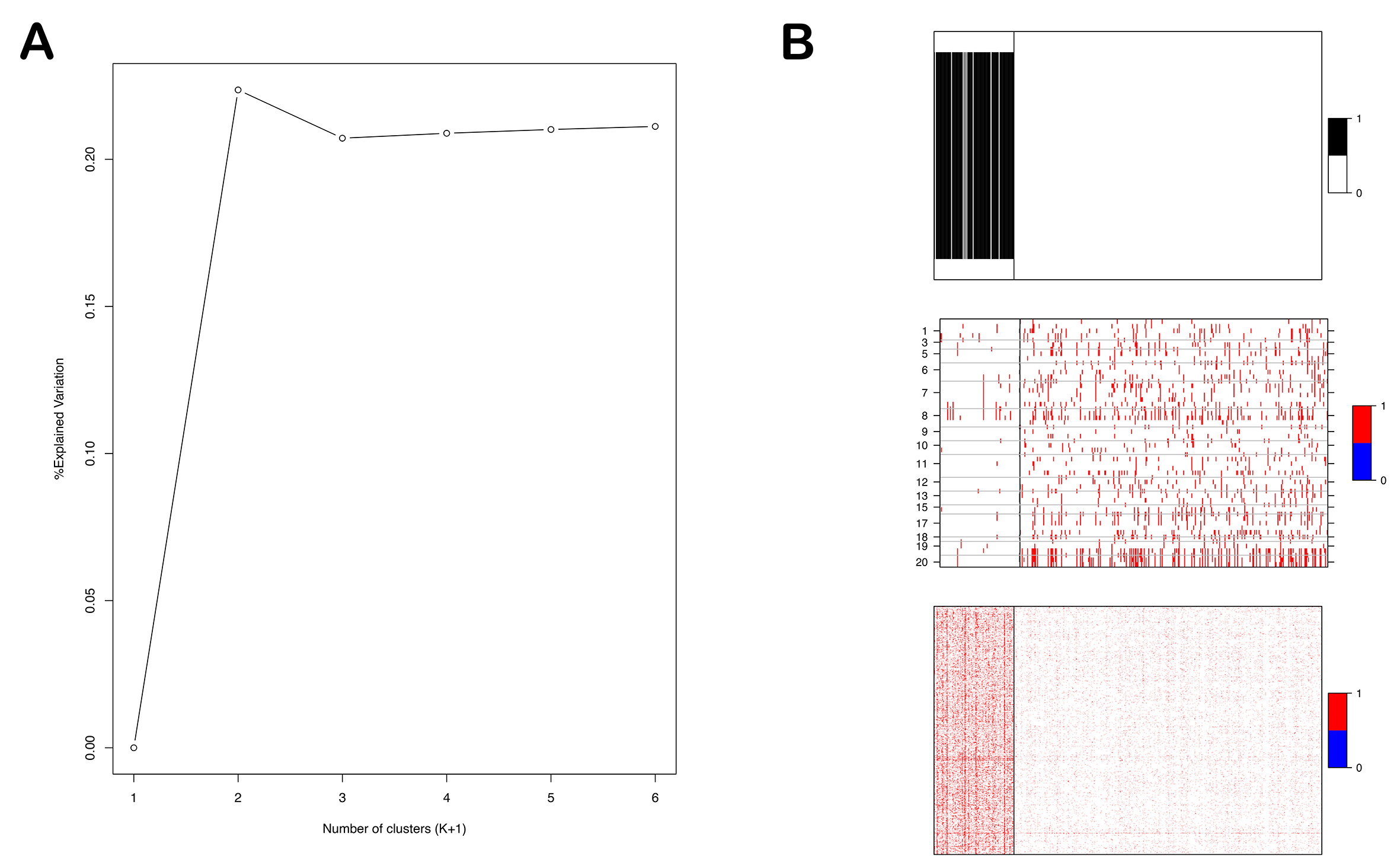

Supplement: Supplementary file 4 [file Image1.tif]

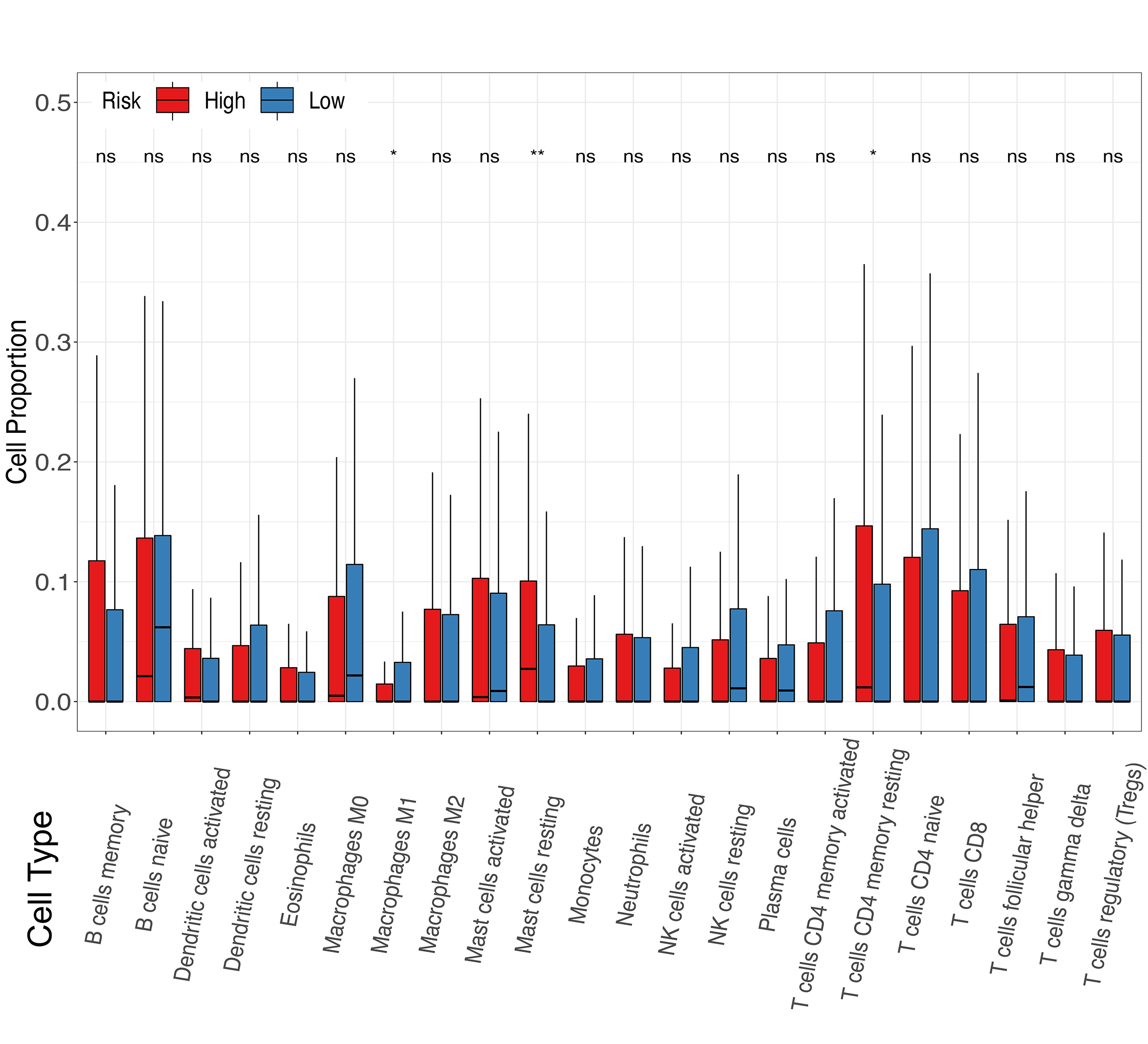

Supplement: Supplementary file 5 [file Image5.tif]
